# The serine hydrolases MAGL, ABHD6 and ABHD12 as guardians of 2-arachidonoylglycerol signalling through cannabinoid receptors

**DOI:** 10.1111/j.1748-1716.2011.02280.x

**Published:** 2012-02

**Authors:** J R Savinainen, S M Saario, J T Laitinen

**Affiliations:** School of Medicine, Institute of Biomedicine/Physiology, University of Eastern Finland (UEF)Kuopio, Finland

**Keywords:** 2-AG hydrolase, ABHD12, ABHD6, endocannabinoid, monoacylglycerol lipase, α/β-hydrolase domain

## Abstract

The endocannabinoid 2-arachidonoylglycerol (2-AG) is a lipid mediator involved in various physiological processes. In response to neural activity, 2-AG is synthesized post-synaptically, then activates pre-synaptic cannabinoid CB1 receptors (CB1Rs) in a retrograde manner, resulting in transient and long-lasting reduction of neurotransmitter release. The signalling competence of 2-AG is tightly regulated by the balanced action between ‘on demand’ biosynthesis and degradation. We review recent research on monoacylglycerol lipase (MAGL), ABHD6 and ABHD12, three serine hydrolases that together account for approx. 99% of brain 2-AG hydrolase activity. MAGL is responsible for approx. 85% of 2-AG hydrolysis and colocalizes with CB1R in axon terminals. It is therefore ideally positioned to terminate 2-AG-CB1R signalling regardless of the source of this endocannabinoid. Its acute pharmacological inhibition leads to 2-AG accumulation and CB1R-mediated behavioural responses. Chronic MAGL inactivation results in 2-AG overload, desensitization of CB1R signalling and behavioural tolerance. ABHD6 accounts for approx. 4% of brain 2-AG hydrolase activity but in neurones it rivals MAGL in efficacy. Neuronal ABHD6 resides post-synaptically, often juxtaposed with CB1Rs, and its acute inhibition leads to activity-dependent accumulation of 2-AG. In cortical slices, selective ABHD6 blockade facilitates CB1R-dependent long-term synaptic depression. ABHD6 is therefore positioned to guard intracellular pools of 2-AG at the site of generation. ABHD12 is highly expressed in microglia and accounts for approx. 9% of total brain 2-AG hydrolysis. Mutations in ABHD12 gene are causally linked to a neurodegenerative disease called PHARC. Whether ABHD12 qualifies as a bona fide member to the endocannabinoid system remains to be established.

Lipid molecules not only serve as building blocks of the central nervous system (CNS) but are being increasingly appreciated as physiological mediators of signal transduction. Molecular components of the lipid signalling machineries show promise as therapeutic targets for various diseases. In contrast with peripheral tissues, brain tissue is enriched in polyunsaturated fatty acids (PUFAs), in particular arachidonic acid (AA, 20:4n-6) and docosahexanoid acid (DHA, 22:6n-3). These long-chain PUFAs are needed for normal brain development and function. Endocannabinoids are endogenous AA-containing lipid mediators involved in the regulation of various physiological and pathophysiological processes, including neurotransmission, mood, appetite, nociception, addiction, inflammation, peripheral metabolism and reproduction (Freund *et al.* 2003, Piomelli 2003, Di Marzo *et al.* 2007, Kano *et al.* 2009). The eCB system consists of two G protein-coupled cannabinoid receptors (CB1R and CB2R), their endogenous activating ligands (the eCBs), as well as enzymes involved in the biosynthesis and inactivation of these ligands. The two well-characterized eCBs, *N*-arachidonoylethanolamine (anandamide, AEA) and 2-arachidonoylglycerol (2-AG), bind to and activate both CB1R and CB2R with somewhat distinct potencies and efficacies. When tested under comparable assay conditions, 2-AG is more potent than AEA and behaves a full agonist at both receptor subtypes (Sugiura *et al.* 2006). In addition, AEA can activate the vanilloid receptor TRPV1, a member of the transient receptor potential family of cation channels that mediates pain sensation (De Petrocellis & Di Marzo 2009). Besides CB1R and CB2R, an orphan G protein-coupled receptor (GPCR), termed GPR55, has been identified and often referred to as the third putative (or atypical) cannabinoid receptor. However, the pharmacology of this receptor is still controversial and an increasing body of evidence suggests that the non-cannabinoid lipid lysophosphatidylinositol, rather than AEA or 2-AG, acts as the cognate agonist of this receptor (Pertwee *et al.* 2010). The purpose of this review is to cover recent research that has advanced our understanding on the physiological regulation of the level and signalling competence of 2-AG through the CBRs. The focus will be on MAGL, ABHD6 and ABHD12, the three serine hydrolases that together account for approx. 99% of 2-AG hydrolysis in the CNS.

## Physiology and logic of the eCB system in the CNS

**Figure 1:**
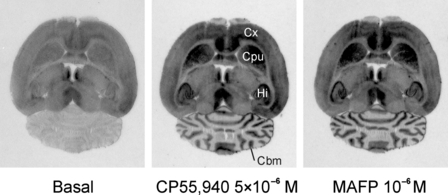
Functional autoradiography reveals wide distribution of CB1R-G_i_ signalling axis in the central nervous system. The method utilizes the radio-labelled GTP analogue [^35^S]GTPγS that incorporates into activated heterotrimeric G proteins in cell membrane following stimulation of G_i_-coupled receptors, either with exogenous or endogenous agonists (Laitinen 2004). The image on the left depicts basal G protein activity in the absence of added agonists and with tonic adenosine A_1_ receptor signal occluded. In the middle panel, CB1Rs were stimulated using the potent synthetic cannabinoid agonist CP55,940. The brain regions with activation of CB1R-Gi axis include the caudate putamen (Cpu), the cerebral cortex (Cx), the hippocampus (Hi), and the molecular layer of cerebellum (Cbm), closely matching CB1R distribution pattern obtained by classical receptor autoradiography (Herkenham *et al.* 1991). In the panel at right, pre-treatment of brain sections with the broad spectrum irreversibly acting serine hydrolase inhibitor methylarachidonylfluorophosphonate (MAFP) results in endogenous 2-arachidonoylglycerol (AG) accumulation and 2-AG-dependent CB1R activity throughout the CB1R-responsive brain regions. Previous studies (Palomäki *et al.* 2007) have demonstrated that (1) the responses to CP55,940 and MAFP are fully blocked by the CB1R-selective antagonist AM251, (2) the MAFP response is not mimicked by selective inhibitor of fatty acid amide hydrolase, ruling our any contribution of AEA, (3) MAFP does not directly activate CB1Rs and 4) MS–GC analysis indicated elevated 2-AG levels in MAFP-treated sections.

**Figure 2:**
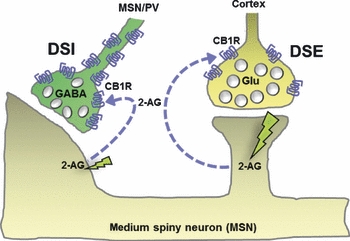
Activity-dependent generation of 2-arachidonoylglycerol (2-AG) for retrograde signalling. Depolarization-induced suppression of inhibition (DSI) is one of the best characterized forms of short-term synaptic plasticity where neuronal activity evokes post-synaptic depolarization via excitatory glutamatergic (Glu) action. This initiates signalling events in the post-synaptic membrane leading to 2-AG biosynthesis and release (dashed arrow). In the retrograde mode of signalling, 2-AG activates pre-synaptic CB1Rs, which are particularly enriched on the inhibitory GABAergic terminals. CB1R-G_i_ signalling results in transient suppression of GABA release. Similar endocannabinoid feedback mechanism operates also at the excitatory synapse where 2-AG-CB1R signalling evokes a cellular response, known as depolarization-induced suppression of excitation (DSE) (adapted and modified from Uchigashima *et al.* (2007)). Note the relative enrichment of CB1Rs on the GABAergic terminals as opposed to the glutamatergic terminals. The relative size of the two sparks illustrates the fact that the stimulation thresholds needed to evoke DSI are generally much lower than those needed to observe DSE (Kano *et al.* 2009, Yoshida *et al.* 2011).

The discovery of CBRs and their endogenous ligands has greatly accelerated research on cannabinoid actions in the brain. Indeed, CB1R is among the most abundantly expressed and widely distributed GPCR in the brain (Herkenham *et al.* 1991) (Fig. 1). CB1R unlikely evolved merely to mediate the ‘bliss’ attributed to delta9-tetrahydrocannabinol (THC), the major psychoactive component of *Cannabis sativa*, nor does nature maintain protein synthesis just for reserve. Instead, the abundance of CB1R in specific types of neurones and its enrichment into pre-synaptic terminals throughout the brain strongly suggests that CB1R evolved specifically to mediate eCB signalling. It is now well established that in response to neural activity, eCBs are produced ‘on demand’ and released from post-synaptic neurones, then activate pre-synaptic CB1Rs in a retrograde manner, resulting in transient and long-lasting reduction of neurotransmitter release at various central synapses (Alger 2002, Freund *et al.* 2003, Piomelli 2003, Kano *et al.* 2009). Such a retrograde signalling mode has established a new concept how diffusible lipid messengers, by encaging their cognate GPCRs, can provide both short- and long-term fine-tuning of synaptic efficacy and neural activity. Electrophysiologists have found robust modulation of synaptic plasticity and thus introduced new terminology, such as depolarization-induced suppression of excitation (DSE), and depolarization-induced suppression of inhibition (DSI), both of which are best explained by short-term retrograde eCB signalling inhibiting synaptic release of glutamate and GABA respectively (Kano *et al.* 2009) (Fig. 2). The presence of molecules of the eCB system, such as the eCBs, CB1R, as well as enzymes involved in eCB metabolism of during neuronal development have been linked to neuronal proliferation, differentiation, migration, axon guidance and synaptogenesis (Bisogno *et al.* 2003, Keimpema *et al.* 2010, Argaw *et al.* 2011). Thus, the eCBs are intimately involved in the physiology of the nervous system.

## Activity-dependent cleavage of membrane phospholipids for retrograde eCB-GPCR signalling

Biosynthesis and release of 2-AG can be induced by either depolarization or activation of G_q_-coupled GPCRs, typically group I metabotropic glutamate receptors (mGluR1 or mGluR5) or M1/M3 muscarinic acetylcholine receptors (Hashimotodani *et al.* 2005, Kano *et al.* 2009). In the hippocampus, 2-AG release is markedly enhanced by simultaneous depolarization and input via G_q_-coupled receptors, a phenomenon where PLC-β1 is suggested to serve as the coincidence detector (Hashimotodani *et al.* 2005). The physiological significance of the coincidence detection is that such a mechanism allows simultaneous sensing of both pre-synaptic (transmitter release) and post-synaptic (membrane depolarization) activities through the Ca^2+^ dependency of PLC-β1 (Hashimotodani *et al.* 2005). In principle, PLC activity generates two second messengers, IP_3_ that triggers Ca^2+^ release from intracellular stores, and diacylglycerol (DAG) that classically activates protein kinase C. In brain regions endowed for eCB signalling, *sn-*2-arachidonoyl-containing DAG species are hydrolysed by *sn*-1-specific lipase (DAGL) to generate 2-AG, the major eCB in brain. Two *sn*-1-specific DAGL isoforms have been cloned, namely DAGLα and DAGLβ (Bisogno *et al.* 2003). The cellular expression of the two isoforms was demonstrated to closely reflect 2-AG biosynthesis and release. During neuronal development, localization of DAGLα and DAGLβ changes from pre- to post-synaptic elements, i.e. from axonal tracts in the embryo to dendritic fields in the adult, closely matching with the developmental changes in need for 2-AG synthesis from the pre- to the post-synaptic compartment (Bisogno *et al.* 2003). Overexpression of DAGLα in mouse neuroblastoma cells results in 2-AG accumulation, whereas knockdown of DAGLα by RNA interference blunts 2-AG production and prevents group I mGluR-stimulated production of this eCB (Jung *et al.* 2007). A docking platform for efficient coupling of glutamatergic signalling to 2-AG biosynthesis is provided by scaffolds such as Homer proteins that allow interaction of group I mGluRs with the PPxxF domain of DAGLα (Jung *et al.* 2007). Interestingly, DAGLβ, which based on recent gene ablation studies is not involved in 2-AG generation for retrograde signalling (Gao *et al.* 2010, Tanimura *et al.* 2010), also lacks the Homer-interacting PPxxF domain (Jung *et al.* 2007). Collectively, these studies suggest that DAGLα has specifically evolved to generate 2-AG for retrograde synaptic signalling. Two recent studies using DAGL-knockout (DAGL-KO) mice have provided strong additional support to this idea. DAGLα-KO mice were found to have marked (up to 80%) reductions in 2-AG levels in brain and spinal cord with concomitant decrease in AA levels, whereas DAGLβ-KO animals exhibited either no (Tanimura *et al.* 2010) or up to 50% reduction (Gao *et al.* 2010) in brain 2-AG levels. Importantly, several forms of retrograde eCB-mediated synaptic suppression, such as DSE and DSI, were absent from the tested brain regions (hippocampus, cerebellum and striatum) of DAGLα-KO mice but appeared intact in DAGLβ-KO mice brains (Gao *et al.* 2010, Tanimura *et al.* 2010). There was no evidence for compensatory changes of DAGLβ in DAGLα-KO mice or vice versa, nor was there evidence for abnormal expression patterns or protein levels of other molecular components of the eCB signalling machinery. Interestingly, eCB control of neurogenesis in the adult hippocampus was compromised in both DAGLα- and DAGLβ-KO mice, as well as in the subventricular zone of DAGLα-KO animals (Gao *et al.* 2010). In peripheral tissues such as the liver, DAGLβ seems to be the major isoform generating 2-AG (Gao *et al.* 2010). Collectively, the genetic studies suggest that DAGLα is the major biosynthetic enzyme generating 2-AG for retrograde signalling. It is interesting to note that in some brain regions highly enriched with the CB1R (such as the substantia nigra), immunohistochemistry reveals only sparse labelling with the DAGLα antibodies (Uchigashima *et al.* 2007, Kano *et al.* 2009, Tanimura *et al.* 2010). This would suggest that alternative biochemical routes for 2-AG biosynthesis (Sugiura *et al.* 2006, Kano *et al.* 2009) are utilized to generate the CB1R-activating eCB in brain regions with sparse DAGLα expression. Further studies with the DAGL-KO mice should shed more light on this issue.

## Synaptic architecture of the eCB signalling machinery for retrograde 2-AG signalling

*In situ* hybridization studies combined with immunohistochemical studies with properly validated antibodies have revealed the ultrastructural localization, molecular composition and synaptic organization of the apparatus needed for 2-AG-mediated retrograde signalling at various central synapses. An excellent review covering details of this topic is available (Kano *et al.* 2009) and therefore only the general aspects are briefly discussed here. The emerging picture is strikingly similar regardless of the brain region and mammalian species studied. At the glutamatergic hippocampal synapses of both rodents and humans, DAGLα is concentrated in dendritic spine heads around the post-synaptic density throughout the hippocampal formation, whereas CB1R is strategically situated at the pre-synaptic axon terminals on the opposite side of synaptic cleft (Katona *et al.* 2006, Kano *et al.* 2009, Ludányi *et al.* 2011). In the striatal medium spiny neurones, DAGLα and the G_q_-coupled GPCRs (mGluR5 and M1) are all enriched on the somatodendritic surface (Uchigashima *et al.* 2007), whereas CB1R localization is pre-synaptic and the receptors are enriched on the GABAergic axon terminals and relatively low abundance on the corticostriatal glutamatergic terminals (Uchigashima *et al.* 2007). Relative enrichment of DAGLα on the post-synaptic membrane at glutamatergic synapses and CB1Rs at the inhibitory terminals instead of excitatory terminals seems to be one hallmark of retrograde eCB signalling at various central synapses (Fig. 2). Such an arrangement likely provides the neuroanatomical and physiological basis for observations where the stimulation thresholds required to evoke DSI are generally much lower than those needed to observe DSE (Kano *et al.* 2009, Yoshida *et al.* 2011).

## Termination of eCB signalling

By analogy to classical neurotransmission, the magnitude and duration of eCB signalling are tightly regulated by the balanced action between the enzymes that synthesize and hydrolyse these lipid messengers. The hydrolysis of eCBs is principally carried out by four enzymes belonging to the metabolic serine hydrolase family. Solid experimental evidence supports the primary role of fatty acid amide hydrolase (FAAH) in the inactivation of AEA both *in vitro* and *in vivo* (Ahn *et al.* 2009). Indeed, comprehensive inactivation of FAAH in rodents either by genetic or pharmacological means results in marked (>10-fold) elevations in brain AEA levels, and as a consequence, CB1R-dependent analgesia in various models of acute and chronic pain (Ahn *et al.* 2009). Importantly, no tolerance of CB1R function and behavioural responses takes place after chronic FAAH inactivation (Ahn *et al.* 2009, Schlosburg *et al.* 2010).

## The serine hydrolases MAGL, ABHD6 and ABHD12 as guardians of 2-AG signalling through the CBRs

The major enzymatic route for 2-AG inactivation in brain is via hydrolysis generating AA and glycerol as the end products. MAGL was the first hydrolase implicated in 2-AG degradation both *in vitro* and *in vivo* (Dinh *et al.* 2002, 2004, Saario *et al.* 2004, Labar *et al.* 2010b). In many tissues and cell types, MAGL is detected both in soluble and membrane preparations. Traditionally, MAGL functions as a key lipolytic enzyme in the mobilization of lipid stores for fuel or lipid synthesis. Accordingly, MAGL was originally purified, and subsequently cloned from the adipose tissue (Karlsson *et al.* 1997, Labar *et al.* 2010b). A recent study has illuminated a pathophysiological, eCB-independent role for MAGL as well. Activity-based protein profiling (ABPP) of various cancer cells has identified MAGL overexpression as the key metabolic switch orchestrating cancer cell malignancy by redirecting lipids from storage sites towards biosynthesis of cancer promoting signalling lipids such as eicosanoids and lysophospholipids (Nomura *et al.* 2010). It was only after discovery of the CBRs and their endogenous ligands that MAGL was linked to the eCB system.

The use of pharmacological inhibitors has provided important initial observations concerning brain 2-AG hydrolases and this issue is briefly touched here. Methylarachidonylfluorophosphonate (MAFP) is among the most potent MAGL inhibitors identified to date (Saario *et al.* 2004, Savinainen *et al.* 2010). MAFP inhibits MAGL irreversibly but lacks selectivity, as it inhibits most members of the metabolic serine hydrolase family. N-arachidonoylmaleimide (NAM) was developed as a potent, cysteine targeting irreversible inhibitor of MAGL-like activity in rat cerebellar membranes (Saario *et al.* 2005). In contrast to MAFP, which comprehensively blocks brain 2-AG hydrolysis (Saario *et al.* 2004), approx. 15% residual activity remained after maximally effective concentration of NAM (Saario *et al.* 2005). We now know that the NAM-resistant activity is caused by the two novel serine hydrolases, namely ABHD6 and ABHD12 (see below). Further studies have indicated that when tested against the brain serine hydrolase proteome, NAM is indeed rather selective for MAGL (Blankman *et al.* 2007). Recently, a new carbamate compound known as JZL184 was introduced as a potent and specific MAGL inhibitor (Long *et al.* 2009). Although the low nanomolar potency of JZL184 has been called into question (Savinainen *et al.* 2010), the selectivity of JZL184 for MAGL over other brain serine hydrolases was convincingly demonstrated using ABPP, a powerful proteomic tool allowing inhibitor activity profiling in complex proteomes (Blankman *et al.* 2007). Thus, NAM and JZL184 can now be used as specific pharmacological tools to dissect out potential role of MAGL as a regulator of 2-AG-CB1R signalling. Indeed, recent studies utilizing these inhibitors both *in vitro* and *in vivo* have provided compelling evidence for the importance of MAGL as the major regulator of not only brain 2-AG levels but also various CB1R-dependent cellular and behavioural responses (Kano *et al.* 2009, Long *et al.* 2009, Pan *et al.* 2009, Straiker *et al.* 2009, Labar *et al.* 2010b).

## Chronic MAGL inactivation results in 2-AG overload, desensitization of CB1R signalling and behavioural tolerance

Long-term disruption of MAGL was achieved either by genetic ablation (MAGL-KO) or chronic treatment with the MAGL-selective inhibitor JZL184 (Chanda *et al.* 2010, Schlosburg *et al.* 2010), and in both studies the general outcome was similar. In contrast to the antinociceptive effects typically observed after acute MAGL inhibition (Kinsey *et al.* 2009, Long *et al.* 2009), animals with chronic MAGL blockade had normal pain responses in several pain models. Moreover, these mice lacked several behavioural responses, such as hypothermia, hypomotility, or catalepsy, typically observed after administration of cannabinoid agonists such as THC. In addition, MAGL-KO mice had decreased body weight, thus resembling the lean phenotype of CB1R-KO mice or animals treated with CB1R antagonists such as rimonabant (Di Marzo *et al.* 2001). Moreover, eCB-mediated short-term synaptic suppression was compromised (Schlosburg *et al.* 2010).

Chronic inactivation of MAGL dramatically decreased the ability of the mice to hydrolyse 2-AG, resulting in massive (>10-fold) increases in brain 2-AG levels. In response to chronic 2-AG overload, compensatory desensitization of brain eCB-CB1R signalling was evident, as both CB1R density (assessed by radioligand-binding studies) and functional responses (assessed by CB1R-dependent G-protein activation assays) were attenuated following chronic MAGL inactivation (Chanda *et al.* 2010, Schlosburg *et al.* 2010). Functional autoradiography revealed that in response to chronic JZL184 administration, desensitization of CB1R signalling was not observed in all brain regions but manifested specifically in regions participating in pain perception and processing (Schlosburg *et al.* 2010). Interestingly, CB1R signalling remained intact in brain regions such as the caudate putamen and globus pallidus. These regions are associated with cannabinoid-induced cataplexy. From the behavioural responses studied, cannabinoid-induced cataplexy also showed minimal cross-tolerance (Schlosburg *et al.* 2010). Collectively, these studies indicate that MAGL is the primary hydrolase responsible for terminating 2-AG signalling in the CNS and that chronic 2-AG overload desensitizes the 2-AG-CB1R signalling axis in specific brain regions, leading to functional and behavioural antagonism of the eCB system.

## 3D-structural insights into MAGL function and substrate recruitment

In 2005, the first homology model of MAGL was presented based on 3D structure of chloroperoxidase from *Streptomyces lividans* as the template (Saario *et al.* 2005). As a result of poor sequence homology between MAGL and chloroperoxidase, the homology model offered limited insights into the overall structure and organization of MAGL protein. However, as the central core of the α/β hydrolase superfamily members is highly conserved, the model offered first insights into the MAGL active site with the catalytic triad (S122-D239-H269), previously identified based on mutagenesis studies (Karlsson *et al.* 1997). The model also suggested that two cysteine residues (C208 and C242) were located within a close distance from the active site and it was suggested that one or both of these cysteines were potential targets of the maleimide-based inhibitor NAM (Saario *et al.* 2005). Site-directed mutagenesis studies have provided experimental support for these predictions (Labar *et al.* 2010a,b).

**Figure 3:**
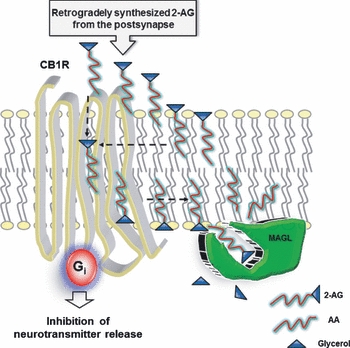
Monoacylglycerol lipase (MAGL) is the prime guardian of 2-arachidonoylglycerol-CB1R (2-AG-CB1R) signalling at the axon terminals. In response to neural activity, 2-AG is produced post-synaptically ‘on demand’ (Fig. 2) and then diffuses across the synapse to activate the pre-synaptic CB1Rs. It is not known whether 2-AG enters the CB1R ligand binding pocket directly from the extracellular space (dashed vertical down arrow) or via lateral diffusion after incorporation into plasma membrane (dashed horizontal left arrow) (Hurst *et al.* 2010). CB1R activation leads to G_i_-mediated short- and long-term inhibition of neurotransmitter release. Ultrastructural localization studies show that MAGL is mainly pre-synaptic and often colocalizes with CB1R in the axon terminals (Kano *et al.* 2009). MAGL is therefore ideally positioned to terminate eCB-CB1R signalling regardless of the source of 2-AG. Inactivation of 2-AG takes place once this lipid messenger dissociates from the receptor and diffuses laterally towards its executor (dashed horizontal right arrow). Highlighted in the illustration are features that emerged from the MAGL 3D structural data (Bertrand *et al.* 2010, Labar *et al.* 2010a). In the illustrated scenario, 2-AG faces its final destiny after dissociating from the CB1R, by entering through the hydrophobic tunnel with its polar glycerol head properly oriented towards the bottom to face the catalytic triad. It is possible that AA may diffuse back to the membrane compartment via the entrance tunnel, whereas glycerol may leave the scene via a separate exit hole (not illustrated) that was disclosed in the MAGL 3D structure.

The crystal structure of MAGL was recently solved and this was achieved independently by two laboratories (Bertrand *et al.* 2010, Labar *et al.* 2010a). Labar *et al.* solved the structure of human MAGL at 2.2 Å resolution and interestingly, MAGL crystallized in this study as a dimer. Bertrand *et al.* resolved the crystal structure of human MAGL both in its apoenzyme form and in complex with the potent covalent inhibitor SAR629. As expected, protein folds conserved in the α/β hydrolase superfamily were present also in the MAGL structure. These include an eight-stranded β-sheet, composed of seven parallel strands and one antiparallel strand, surrounded by α helices. Moreover, a flexible cap domain covers the structurally conserved β-sheet and a lid domain guards the entrance of a relatively large, occluded hydrophobic tunnel (approx. 25 Å in length and approx. 8 Å in width). The active site is buried at the bottom of the tunnel. For a detailed structural analysis and discussion (both of which are beyond the scope of this review), the the original reports are referred to the reader (Bertrand *et al.* 2010, Labar *et al.* 2010a). However, in the context of physiological regulation of 2-AG-CB1R signalling, some points emerging from the 3D structures deserve further discussion here. For instance, it is likely, although not experimentally proven, that MAGL orientation is especially well-suited to recruit its substrate directly from the lipid membrane (Fig. 3). The 3D structure also points to the possibility that the glycerol formed in 2-AG hydrolysis might leave the scene through a narrow hydrophilic tunnel, referred to as an ‘exit hole’ (Bertrand *et al.* 2010). Docking studies with 2-AG and 1(3)-AG indicate that the two isomers bind to the active site in similar manner (Bertrand *et al.* 2010), thus providing the molecular basis for earlier observations that MAGL hydrolyses the two isomers at similar rates (Saario *et al.* 2004).

## ABHD6 as a guardian of 2-AG-CBR signalling at the site of eCB generation

**Figure 4:**
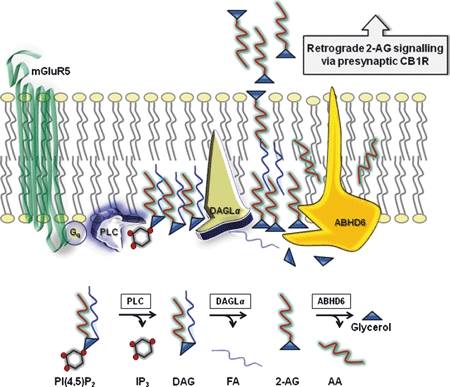
ABHD6 is strategically positioned to regulate 2-AG levels at the site of its generation. ABHD6 is an integral membrane protein localizing to the post-synaptic neuronal membrane. The PLC-DAGLα pathway cleaves the membrane phospholipid PIP_2_ to generate 2-AG for retrograde signalling through pre-synaptic CB1Rs (Fig. 2). Immunohistochemical studies indicate that in neurones ABHD6 localizes to sites of 2-AG generation, including post-synaptic dendrites of principal glutamatergic neurones as well as some GABAergic interneurones (Marrs *et al.* 2010). 3D structure of ABHD6 has not been resolved but the catalytic triad (depicted as mouth) is predicted to face the cytosol/intracellular membrane (Blankman *et al.* 2007).

ABHD6 is a newly discovered post-genomic protein and relative little is known on its physiological functions. High expression of ABHD6 has been reported in certain forms of tumours suggesting that ABHD6 might serve a new diagnostic marker of these tumours (Li *et al.* 2009, Max *et al.* 2009). However, its knockdown in cancer cells did not inhibit tumour cell growth (Max *et al.* 2009). Based on hydropathy analysis and biochemical studies, ABHD6 appears to be an integral membrane protein (Blankman *et al.* 2007) that possesses typical α/β-hydrolase family fingerprints such as the lipase motif (GHSLG) and a fully conserved catalytic triad (postulated amino acid residues S246-D333-H372). The active site is predicted to face cell interior. Such an orientation suggests that ABHD6 is well suited to guard the intracellular pool of 2-AG (Fig. 4). A recent study (Marrs *et al.* 2010) provided the first evidence to link ABHD6 as a bona fide member to the eCB system by demonstrating that ABHD6 controls the accumulation and efficacy of 2-AG at the CBRs. In neurones, ABHD6 was detected both at mRNA and protein level and its pharmacological inhibition led to activity-dependent accumulation of 2-AG in neuronal cultures. In the mouse cortex, ABHD6 localization in neurones was mainly post-synaptic, often juxtaposed with the pre-synaptic CB1Rs. ABHD6 was expressed also in many principal glutamatergic neurones, some GABAergic inteneurones, as well as astrocytes but not in resident microglia (Marrs *et al.* 2010). However, in a microglial cell line (BV-2), ABHD6 was enriched in mitochondrial fraction and its knockdown reduced the hydrolysis of 2-AG in intact cells with concomitant sensitization of 2-AG-stimulated and CB2R-dependent cell migration. In murine cortical slices, pharmacological ABHD6 inhibition facilitated the induction of CB1R-dependent long-term synaptic depression by otherwise subthreshold stimulation. Interestingly, CB1R-dependent short-term synaptic depression (DSI or DSE) remained unaltered following ABHD6 inhibition. The postulated physiological role of ABHD as a regulator of 2-AG levels at the site of production of this eCB is schematically illustrated in Figure 4.

## ABHD12 – a microglial 2-AG hydrolase with poorly characterized function

**Figure 5:**
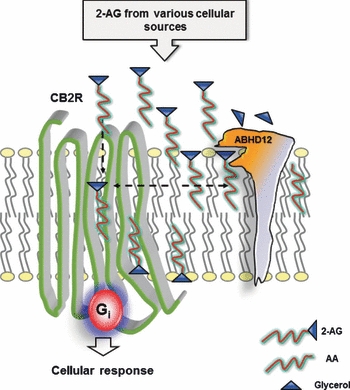
Hypothetical scheme where ABHD12 functions as an ectohydrolase regulating extracellular 2-AG levels and CB2R signalling in microglia. ABHD12 is predicted to be an integral membrane protein (Blankman *et al.* 2007) and in the brain its mRNA is highly expressed in microglia. ABHD12 transcripts are also found in related cell types (macrophages, osteoclasts) and this hydrolase is therefore potentially positioned to guard 2-AG-CBR signalling in these cells. Although ABHD12 hydrolyses 2-AG, it is not currently known whether it utilizes other substrates and serve other functions. At the bulk brain level, ABHD12 accounts for approx. 9% of total 2-AG hydrolase activity. The 3D structure of ABHD12 is not available but the active site (depicted as a mouth digesting 2-AG) is predicted to face the luminal/extracellular side. ABHD12 may thus function as an ectohydrolase. In the hypothetical scenario, ABHD12 guards extracellular 2-AG levels and subsequent CB2R activity. 2-AG may enter the CB2R ligand binding pocket directly from the extracellular space (dashed vertical down arrow), or as suggested based on molecular dynamics stimulation (Hurst *et al.* 2010), via lateral diffusion after incorporation into the membrane (dashed horizontal left arrow). CB2R activation leads to G_i_-mediated cellular responses such as proliferation and migration (Cabral *et al.* 2008). Inactivating ABHD12 mutations have been casually linked to a neurodegenerative condition known as PHARC (polyneuropathy, hearing loss, ataxia, retinitis pigmentosa and cataract) (Fiskerstrand *et al.* 2010). It is not currently known whether this condition is directly linked to the endocannabinoid system.

Like ABHD6, ABHD12 is a recently identified post-genomic protein with poorly defined physiological function. Its potential role as a brain 2-AG hydrolase was revealed using ABPP with mouse brain proteome and it was estimated that at the bulk brain level ABHD12 accounts for approx. 9% of total 2-AG hydrolase activity (Blankman *et al.* 2007). Currently, 2-AG is the only known substrate for ABHD12 but it is possible that the enzyme utilizes also other substrates. As far as we are aware, 2-AG hydrolase activity is the only feature so far potentially linking ABHD12 to the eCB system. Based on hydropathy analysis and biochemical data, ABHD12 appears to be an integral membrane protein whose active site is predicted to face the lumen/extracellular space (Blankman *et al.* 2007). Typical α/β-hydrolase domain protein fingerprints, including the lipase motif (GTSMG) and catalytic triad (predicted amino acid residues S148-D278-H306), are fully conserved both in rodent and human ABHD12 primary structure. It was recently reported that mutations in the ABHD12 gene that are predicted to compromise catalytic activity severely, are causally linked to a neurodegenerative disease called PHARC (polyneuropathy, hearing loss, ataxia, retinitis pigmentosa, and cataract) (Fiskerstrand *et al.* 2010). Based on this surprising finding, the authors suggested that ABHD12 must perform essential physiological functions in the nervous system and that PHARC may serve as a human ABHD12 KO model (Fiskerstrand *et al.* 2010). However, tissue 2-AG levels and metabolism were not explored in this study and thus the possible connection to the eCB system clearly requires further studies. Interestingly, ABHD12 transcripts are highly expressed in various brain regions and specifically in microglia, but are also abundant in related cell types such as macrophages and osteoclasts (Fiskerstrand *et al.* 2010). There is also increasing appreciation for the potential role of the eCB system, and microglial CB2Rs in particular, as regulators of immune function in the CNS (Cabral *et al.* 2008). Thus, as schematically illustrated in Figure 5, ABHD12 appears to be well suited to guard 2-AG-CB2R signalling in brain microglia as well as in related cell types of peripheral tissues.

## Concluding ideas and possible directions for future research

Delicate physiological regulatory mechanisms have evolved to maintain the balance between the ‘on demand’ biosynthesis and degradation of the signalling competent pool of 2-AG in the CNS. Significant progress has been made during the last few years in dissecting out details of the synaptic architecture and molecular components of the eCB system intimately involved in the physiology and function of the nervous system. Recent milestones include the elucidation of MAGL crystal structure, as well as the availability of selective pharmacological and genetic tools to specifically target key enzymes involved in the generation (DAGLα) and degradation (MAGL) of 2-AG. Insights into MAGL crystal structure open new avenues to exploit MAGL function in further detail. From the perspective of rational drug design, the shape of the hydrophobic tunnel leading to the catalytic site suggests ‘a high druggability of the protein’ (Bertrand *et al.* 2010), offering possibilities for further development of potent and specific MAGL inhibitors. However, one may ask the question whether there is further need for such inhibitors as potential therapeutics. The answer might be yes if we consider the recently disclosed pathophysiological role of MAGL in promoting cancer cell malignancy (Nomura *et al.* 2010). In addition, specific pharmacological tools are needed to explore MAGL function further. On the other hand, the answer might be no if we consider MAGL inhibitors as potential therapeutics to alleviate pain, for example. Direct CBR1 agonists like THC produce analgesia in various pain models but their therapeutic use is limited because of undesired psychotropic effects. Prolonging and amplifying the eCB tone by inhibiting their enzymatic metabolism has therefore emerged as an alternative strategy to manipulate the eCB system for possible clinical benefit (Lambert & Fowler 2005, Mackie 2006, Hohmann 2007, Saario & Laitinen 2007, Petrosino & Di Marzo 2010). We have just learnt that chronic MAGL inhibition leads to 2-AG overload and functional antagonism of the eCB system, both at the molecular and behavioural level (Lichtman *et al.* 2010). Would partial MAGL inhibition result in pain relief without desensitization of the eCB system? Might FAAH be a better molecular target of the eCB system for pain relief? This reasoning is supported by findings that the analgesic effects of a FAAH inhibitor persist after long-term administration and no apparent desensitization of CB1R function takes place after chronic FAAH inactivation (Ahn *et al.* 2009, Schlosburg *et al.* 2010). We know very little on the physiological or pathophysiological roles of ABHD6 and ABHD12. The postulated causal link between ABHD12 mutations and the neurodegenerative disease PHARC should stimulate further research that will clarify whether ABHD12 is a molecular component of the eCB system. As always, new important findings tend to raise more questions than to provide final answers. This in mind it appears that researchers on the eCB field will be busy also during the forthcoming years.

## Conflict of interest

There is no conflict of interest.
